# Neoplasm Risk in Patients With Rheumatoid Arthritis Treated With Fostamatinib: A Systematic Review and Meta-analysis

**DOI:** 10.3389/fphar.2022.768980

**Published:** 2022-03-02

**Authors:** Yuehong Chen, Huan Liu, Yunru Tian, Zhongling Luo, Geng Yin, Qibing Xie

**Affiliations:** Department of Rheumatology and Immunology, West China Hospital, Sichuan University, Chengdu, China

**Keywords:** fostamatinib, rheumatoid arthritis, neoplasm, meta-analysis, systematic review

## Abstract

**Objective:** This study aimed to assess neoplasm risk in patients with rheumatoid arthritis (RA) treated with fostamatinib.

**Methods:** Studies were collected from electronic databases of OVID Medline, OVID EMBASE, Cochrane Central Register of Controlled Trials, and Web of Science. We included studies that reported neoplasms in patients with RA treated with fostamatinib. Study selection was repeated by two reviewers based on the study selection criteria. Data were collected and methodological quality assessment was performed. Data were pooled using the Peto odds ratio (OR) with a 95% confidence interval (CI). Subgroup analyses of the fostamatinib dose, trial duration, neoplasm nature, and neoplasm-originating systems were conducted. A funnel plot was used to estimate publication bias, and sensitivity analysis was performed to test the robustness of the results.

**Results:** Seven trials involving 4,971 participants showing low to moderate risk of bias were included. Compared with the placebo, fostamatinib use was not associated with the risks of overall neoplasms (Peto OR = 2.62, 95%CI 0.97–7.10), malignant neoplasms (Peto OR = 3.08, 95%CI 0.96–9.91), or benign neoplasms (Peto OR = 1.71, 95%CI 0.26–11.36). Nevertheless, compared with the placebo, a longer duration of fostamatinib use had a higher risk of malignant neoplasms (Peto OR = 4.49, 95%CI 1.03–19.60) at 52 weeks. As for malignant neoplasms in the digestive system, lower doses of fostamatinib reduced the neoplasm risk (100 mg bid vs 150 mg qd: Peto OR = 0.06, 95%CI 0.01–0.59). Sensitivity analysis showed no significant differences in the effective trends, and no publication bias was found.

**Conclusion:** Fostamatinib is not associated with the risks of overall neoplasms as compared to placebo. Nevertheless, a longer duration of fostamatinib use may be associated with a risk of malignant neoplasms and higher doses of fostamatinib may increase malignant neoplasms in the digestive system. Further well-planned cohort studies with a larger study population are needed to elucidate these outcomes.

**Systematic Review** **Registration:** PROSPERO (CRD42020202121).

## 1 Introduction

Rheumatoid arthritis (RA) is an autoimmune disease characterized by persistent synovitis formation, systematic inflammation, and autoantibodies presence, leading to bone and cartilage damage if not treated appropriately (Scott et al., 2010; Smolen et al., 2016). The risk factors for RA include susceptibility genes, environmental factors (e.g., oral microbiome, smoking, periodontitis, and microbiome), epigenetic modifications, and posttranslational modifications (e.g., methylation, acetylation, and citrullination). RA is associated with several comorbidities, such as cancer and cardiovascular diseases, which are risk factors for increased mortality (Dougados, 2016; Widdifield et al., 2018).

Compared with the general population, patients with RA are associated with increased risks of overall malignant neoplasms [standardized incidence ratio (SIR) = 1.05, 95% confidence interval (CI) (1.01–1.09)], lymphoma [2.08, 95%CI (1.80–2.39)], lung cancer [1.63, 95%CI (1.43–1.87)], and malignant melanoma [1.23, 95%CI (1.01–1.49)] (Smitten et al., 2008; Simon et al., 2015; De Cock and Hyrich, 2018). The high inflammatory activity of RA is a major risk factor for developing lymphomas (Baecklund et al., 2006). Compared with low disease activity, moderate and high inflammatory disease activity increase the risk of developing lymphomas by 8-fold (odds ratio [OR] = 7.7, 95%CI 4.8–12.3) and 70-fold (OR = 71.3, 95%CI 24.1–211.4), respectively. Therefore, achieving remission of disease activity or maintaining low disease activity is the treatment target (Smolen et al., 2016).

Spleen tyrosine kinase (Syk), a non-receptor protein tyrosine located in the cytoplasm, plays a fundamental role in the activation of the B-cell receptor, which is necessary for B-cell development, proliferation, and survival. Thus, pharmacological targeting of Syk is effective in affecting the signal transduction of B-cell receptors, leading to cell apoptosis and inhibition of the activation and migration of B-cells, which are therapeutic targets for B-cell dominant diseases, such as chronic lymphocytic leukemia, B-cell malignancies, and autoimmune disorders (Buchner et al., 2010; Hoellenriegel et al., 2012). Fostamatinib is a Syk inhibitor, and R406 is the active metabolite of fostamatinib, which has been reported to effectively treat RA (Kunwar et al., 2016; Kang et al., 2019). Nevertheless, fostamatinib is reported to have an increased risk of infection, diarrhea, hypertension, neutropenia, and hypertransaminasaemia (Salgado et al., 2014; Kunwar et al., 2016; Kang et al., 2019; Chen et al., 2021). Whether the use of fostamatinib is associated with an increased risk of malignancy remains unknown. Therefore, we performed a systematic review and meta-analysis by including all the available evidence to assess neoplasm risk in patients with RA treated with fostamatinib.

## 2 Methods

### 2.1 Setting

This systematic review and meta-analysis was conducted to investigate the neoplasm risk in patients with RA treated with fostamatinib and reported based on the preferred reporting items for systematic reviews and meta-analyses guidelines (Moher et al., 2009). This study was registered in PROSPERO with registration number CRD42020202121.

### 2.2 Eligibility Criteria

The inclusion criteria were set based on the PICO principle: P (patients) were RA patients; I (intervention) was fostamatinib, regardless of the dose and usage; C (comparison) was placebo, other treatment, or different doses of fostamatinib; O (outcomes) were neoplasms regardless of the neoplasm nature (malignant or benign); in addition, we also included s (study designs) for randomized controlled trials (RCTs), cohort studies, or case-control studies.

A study was excluded if it was a duplicate, commentary, conference abstract, and or did not have relevant outcomes.

### 2.3 Search Strategy

Electronic databases of OVID Medline, OVID EMBASE, Web of Science, and Cochrane Central Register of Controlled Trials were searched on 3 July 2020, using both MeSH terms and key words without language limitations. Search terms included “rheumatoid arthritis” and “fostamatinib”. Detailed search strategy can be found in the supplemental file or the published study (Chen et al., 2021). Manual searches of reference lists of included studies and clnicaltrials.gov was also performed to identify potentially eligible studies.

### 2.4 Study Selection

Study selection was performed independently and repeated by a pair of reviewers (CYH, LH, and TYR), which was managed by Microsoft Office Access 2013. After preliminary screening by titles and abstracts, full texts were read based on the study selection criteria. Reference lists of included studies and published reviews and the clinicaltrials.gov website were manually checked. Any disagreement was resolved via discussion or judged by a third reviewer, if necessary.

### 2.5 Data Extraction

Data was collected independently and repeated by two authors (CYH, TYR, and LZL) on trial registration number, publication date or release date, trial duration-from the trial beginning to the time assessing neoplasm incidence, treatment information, number of neoplasms, and number of participants. Any disagreement was resolved via discussion or judged by a third reviewer, if necessary.

### 2.6 Methodological Quality Assessment

The Cochrane Collaboration tool was used to assess the risk of bias of included RCTs (JPT and So, 2011). Which focused on the items of random sequence generation, allocation concealment, blinding of participants and personnel, blinding of outcome assessment, incomplete outcome data, and selective reporting. For each item, if the answer was yes and correctly described, the assessment was low risk; if the answer was yes but lacked detailed description, the assessment was unclear; if the answer was yes but with the inappropriate method or if the method was not performed, the assessment was high risk. The risk of bias of the included studies was judged based on overall evidence. Methodological quality assessment was performed by two reviewers (CYH and LH), and any disagreement was resolved via discussion or judged by a third reviewer, if necessary. Quality assessment for cohort and case-control studies was not described here, as no such study design was included.

### 2.7 Data Analyses

RevMan software (version 5.1.3) was used to analyze the data. The effect size of the meta-analysis was estimated using the Peto OR with 95% CIs, considering the very low event. I^2^ and heterogeneity *p*-value at the level of 0.1 was used to assess the clinical diversity, and I^2^ had values of 25, 50, and 75% indicating low, moderate, and high heterogeneity, respectively, as recommended by the Cochrane Handbook (Higgins et al., 2003). Pre-set subgroup analyses by fostamatinib dose, trial duration, neoplasm nature, and neoplasm-originating systems were conducted. Sensitivity analysis using the Mantel-Haenszel random effect model was performed to test the robustness of the results. Publication bias was assessed using funnel plots.

Classification of neoplasm-originating sites and systems was based on the 10^th^ version of the International Classification of Diseases (ICD10) (WHO), including malignant neoplasms (C00-C97), benign neoplasms (D10-D36), bone and articular cartilage (C40-C41), ill-defined, secondary, and unspecified sites (C76-C80), digestive organs (C15-C26), and urinary tract (C64-C68).

### 2.8 Patient and Public Involvement

As this study is a systematic review and meta-analysis, no ethical concerns or patients were involved.

## 3 Results

### 3.1 Study Selection

**FIGURE 1:**
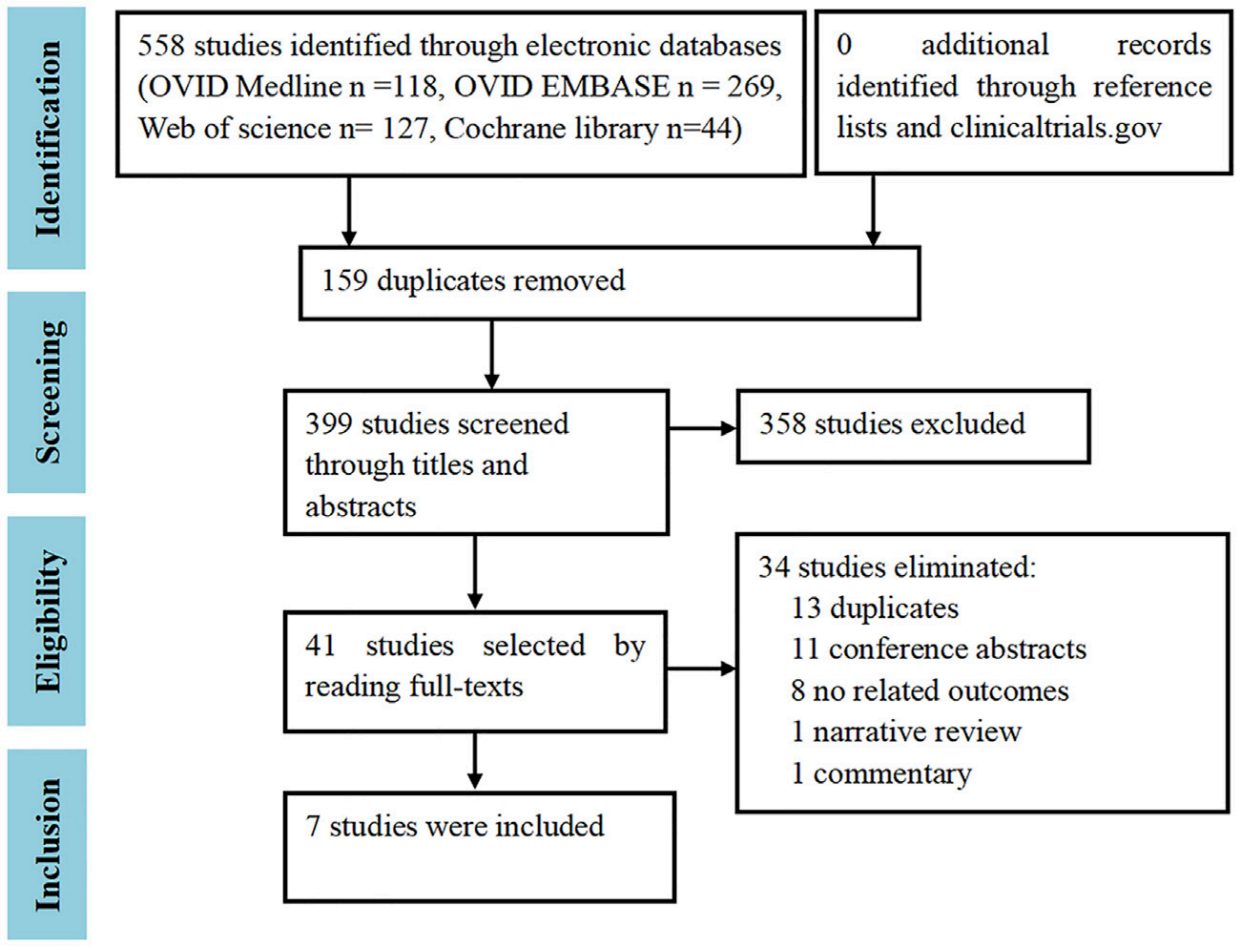
Study selection flowchart. Adapted from Chen et al. (2021).

A total of 558 citations were identified from the OVID Medline (*n* = 118), OVID EMBASE (*n* = 269), Web of Science (*n* = 127), and Cochrane Library (*n* = 44). After excluding duplicates (*n* = 159), irrelevant studies (*n* = 358) screened by titles and abstracts, and studies without relevant outcomes (*n* = 34) by reading the full texts, finally, seven trials (Evaluation of Effectivene, 1197a; Evaluation of Effectivene, 1197b; Evaluation of Effectivene, 1197c; Evaluation of Efficacy an, 1264; Treatment of Arthritis Wi, 2633; Efficacy and Safety Study; Evaluation of Long-term S, 1242) that enrolled a total of 4,971 RA patients were included (Figure 1). No additional studies were included by the manual checking. No cohort or case-control studies were included.

### 3.2 Characteristics of Included Studies

The trial duration was a median (range) of 26 (12–109) weeks. The number of participants varied from 189 to 1912, with a median of 457. About 82.7% (4,113/4,971) of the participants were women. The age was around 50 years, ranging from 50 to 53 years. The common dosage of fostamatinib was 100 mg twice daily or 150 mg once daily, which were taken orally (Table 1).

**TABLE 1:** Characteristics of included RCTs.

Included RCTs	Year of results first posted	Trial duration	NO. of participants (female/male)	Age (years, mean ± SD)	Treatments (no. of participants)
NCT01197521 (Evaluation of Effectivene, 1197a)	2014	52 weeks	918 (770/148)	52 ± 12.0	Fostamatinib 100 mg bid (310)
					Fostamatinib 100 mg bid (4 weeks) then 150 mg qd (304)
					placebo (24 weeks) then fostamatinib 100 mg bid (304)
NCT01197534 (Evaluation of Effectivene, 1197b)	2014	52 weeks	908 (742/166)	53 ± 11.9	Fostamatinib 100 mg bid (308)
					Fostamatinib 100 mg bid (4 weeks) then 150 mg qd (298)
					placebo (24 weeks) then fostamatinib 100 mg bid (302)
NCT01197755 (Evaluation of Effectivene, 1197c)	2014	24 weeks	322 (261/61)	53 ± 12.3	Fostamatinib 100 mg bid (105)
					Fostamatinib 100 mg bid (4 weeks) then 150 mg qd (108)
					placebo (109)
NCT01264770 (Evaluation of Efficacy an, 1264)	2014	24 weeks	265 (210/55)	50 ± 11.8	Fostamatinib 100 mg bid (54)
					Fostamatinib 100 mg bid (4 weeks) then 100 mg qd (57)
					Fostamatinib 100 mg bid (4 weeks) then 150 mg qd (48)
					placebo (6 weeks) then fostamatinib 100 mg bid (27)
					placebo (6 weeks) then fostamatinib 100 mg bid (4 weeks) then 150 mg qd (25)
					Adalimumab 40 mg every 2 weeks (54)
NCT00326339 (Treatment of Arthritis Wi, 2633)	2008	12 weeks	189 (164/25)	52.1 (20–75), median	Fostamatinib 50 mg bid (46)
					Fostamatinib 100 mg bid (49)
					Fostamatinib 150 mg bid (47)
					placebo (47)
NCT00665925 (Efficacy and Safety Study)	2016	26 weeks	457 (390/67)	52.5 ± 12.8	Fostamatinib 100 mg bid (152)
					Fostamatinib 150 mg qd (152)
					placebo (153)
NCT01242514 (Evaluation of Long-term S, 1242)	2014	109 weeks	1912 (1576/336)	53 ± 11.8	Fostamatinib 100 mg qd (212)
					Fostamatinib 100 mg bid (1343)
					Fostamatinib 150 mg qd (357)

Bid = twice a day; qd = once a day.

### 3.3 Methodological Quality

**FIGURE 2:**
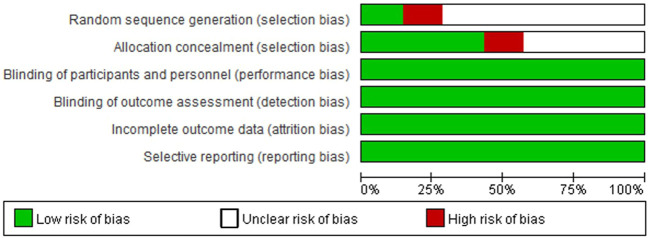
Risk of bias graph. Adapted from Chen et al. (2021).

**FIGURE 3:**
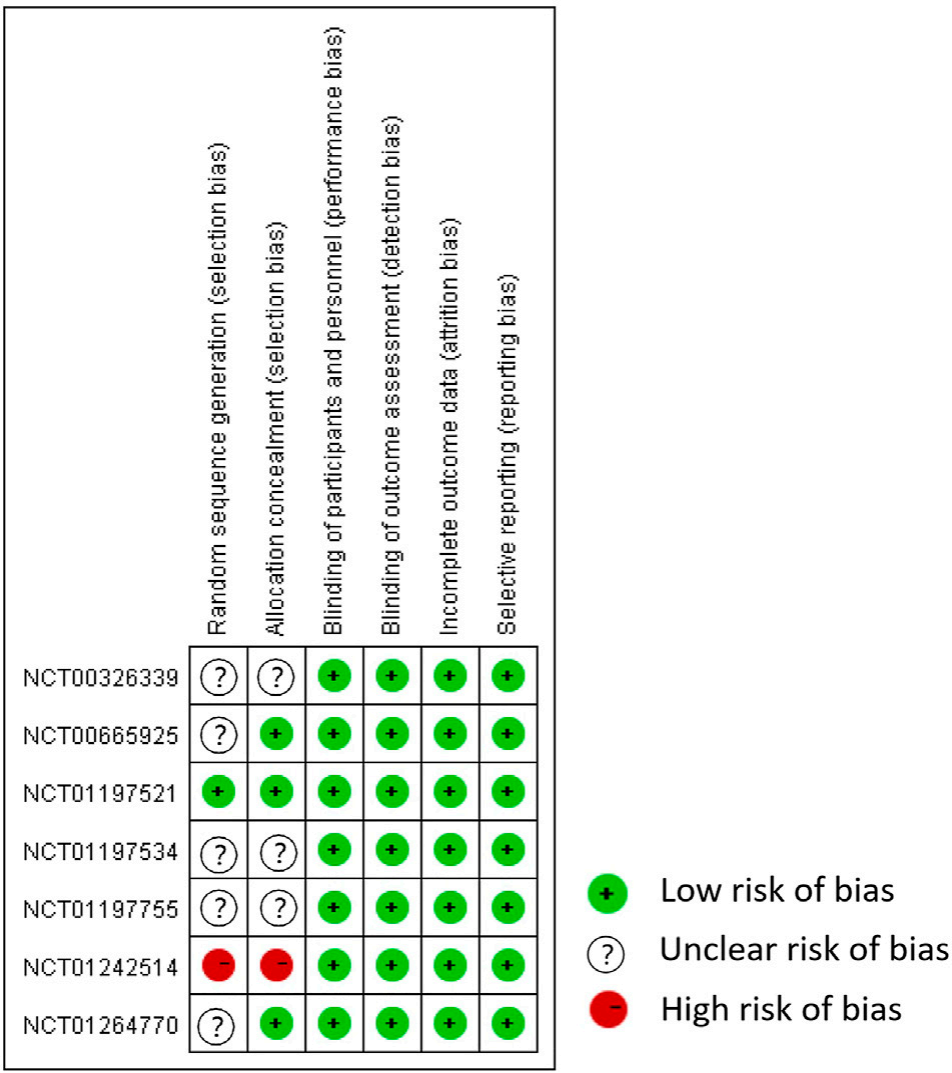
Risk of bias summary. Adapted from Chen et al. (2021).

Only one study correctly reported methods of random sequence generation, three studies correctly reported allocation concealment, and all trials performed blinding of participants, personnel, and outcome assessment, reported complete outcome data, and did not selectively report outcome data (Figures 2, 3). Overall, the risk of bias in the included trials was low to moderate.

### 3.4 Sensitivity Analysis and Publication Bias

**FIGURE 4:**
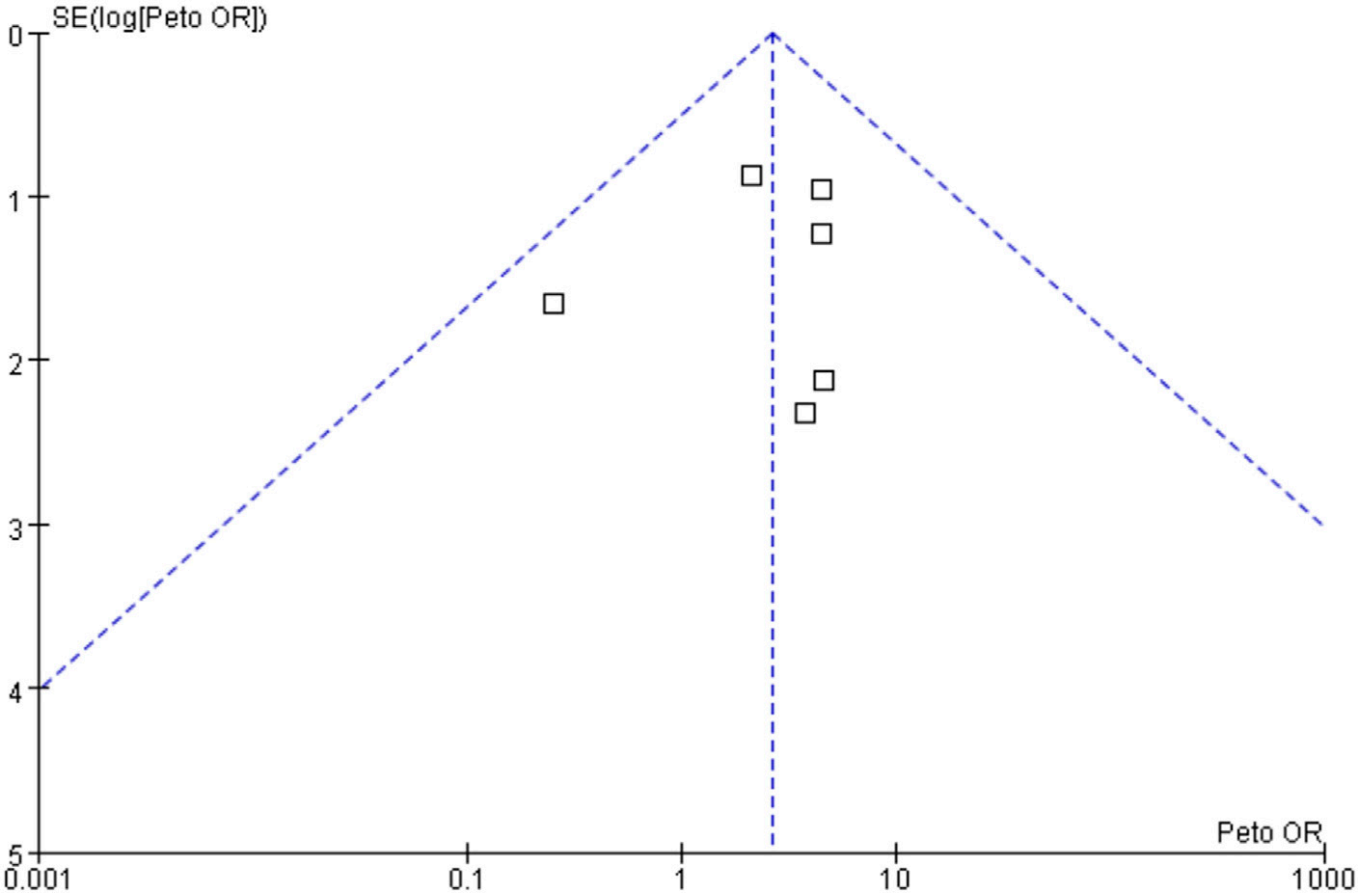
Publication bias based on overall neoplasms in patients treated with fostamatinib versus placebo.

Different statistical methods were used, the Mantel-Haenszel random effect model vs. Peto fixed-effect model, to conduct sensitivity analysis to test the robustness of the results, which did not change the effect direction (data not shown). Publication bias, taking the data from overall neoplasms in patients treated with fostamatinib vs. placebo for an example, was assessed using a funnel plot, and the results showed that the funnel plot was symmetrical; thus, publication bias was not likely to occur (Figure 4).

### 3.5 Main Outcomes

#### 3.5.1 Overall Neoplasms

**FIGURE 5:**
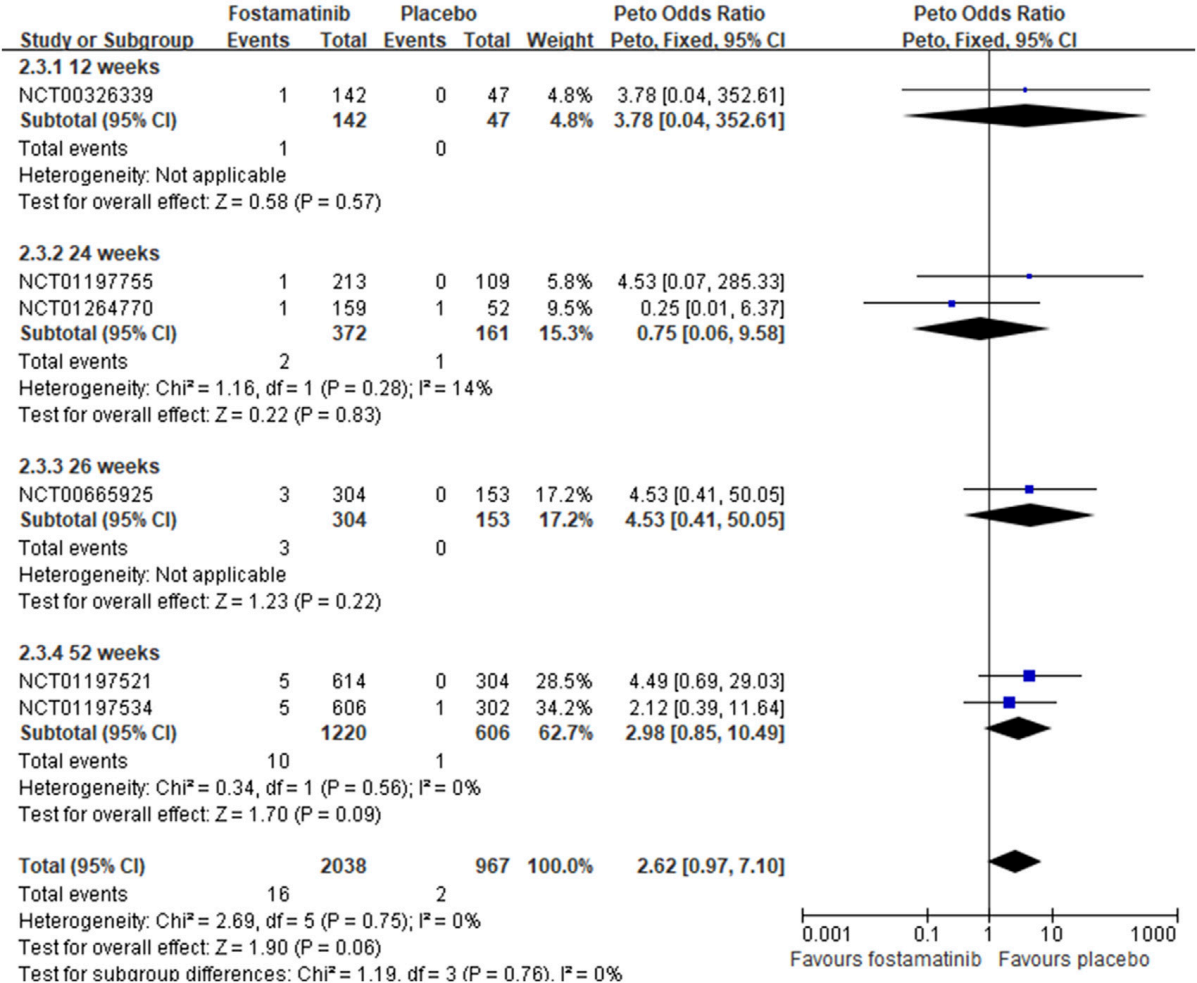
Forest plot of overall neoplasms based on fostamatinib versus placebo.

In comparing fostamatinib vs. placebo, a total of six trials reported 16 neoplasms in 2,038 RA patients treated with fostamatinib, which did not increase the neoplasm risk compared with the placebo of two neoplasms in 967 RA patients (Peto OR = 2.62, 95%CI 0.97–7.10, I^2^ = 0, 16/2038 vs. 2/967) (Table 2; Figure 5). Longer durations of fostamatinib use also did not increase the neoplasm risk without clinical diversity across the studies (Peto OR = 3.78, 95%CI 0.04–352.61, 1/142 vs. 0/47 for 12 weeks; Peto OR = 0.75, 95%CI 0.06–9.58, 2/372 vs. 1/161 for 24 weeks; Peto OR = 4.53, 95%CI 0.41–50.05, 3/304 vs. 0/153 for 26 weeks; Peto OR = 2.98, 95%CI 0.85–10.49, 10/1220 vs. 1/606 for 52 weeks) (Table 2; Figure 5).

**TABLE 2:** Pooled data of neoplasms in RA patients.

Comparisons	No of study	Fostamatinib		Comparator		Heterogeneity		Peto OR, 95% CI
		No of cancer	No of participants	No of cancer	No of participants	I2	P	
Overall neoplasms								
Fostamatinib vs PBO	6	16	2038	2	967	0	0.75	2.62 [0.97, 7.10]
12 weeks	1	1	142	0	47	—	—	3.78 [0.04, 352.61]
24 weeks	2	2	372	1	161	14	0.28	0.75 [0.06, 9.58]
26 weeks	1	3	304	0	153	—	—	4.53 [0.41, 50.05]
52 weeks	2	10	1220	1	606	0	0.56	2.98 [0.85, 10.49]
Fostamatinib 100 mg bid vs. 150 mg qd	7	25	2954	12	1339	35	0.17	0.81 [0.39, 1.69]
12 weeks	1	0	49	0	47	—	—	—
24 weeks	2	0	186	2	181	0	0.96	0.13 [0.01, 2.08]
26 weeks	1	2	152	1	152	—	—	1.96 [0.20, 18.95]
52 weeks	2	7	1224	4	602	82	0.02	0.86 [0.24, 3.02]
109 weeks	1	16	1343	5	357	—	—	0.84 [0.29, 2.42]
Fostamatinib 100 mg bid vs. 100 mg qd	2	16	1424	0	269	—	—	3.22 [0.77, 13.52]
Malignant neoplasms								
fostamatinib vs PBO	6	12	2038	1	967	6	0.38	3.08 [0.96, 9.91]
12 weeks	1	1	142	0	47	—	—	3.78 [0.04, 352.61]
24 weeks	2	1	372	1	161	68	0.08	0.36 [0.02, 7.76]
26 weeks	1	2	304	0	153	—	—	4.51 [0.24, 85.33]
52 weeks	2	8	1220	0	606	0	1	**4.49 [1.03, 19.60]**
Fostamatinib 100 mg bid vs. 150 mg qd	7	19	2954	7	1339	39	0.16	1.09 [0.45, 2.60]
12 weeks	1	0	49	0	47	—	—	—
24 weeks	2	0	186	1	181	—	—	0.14 [0.00, 7.02]
26 weeks	1	2	152	0	152	—	—	7.44 [0.46, 119.46]
52 weeks	2	6	1224	3	602	72	0.06	0.98 [0.24, 3.96]
109 weeks	1	11	1343	3	357	—	—	0.97 [0.27, 3.54]
Fostamatinib 100 mg bid vs. 100 mg qd	2	11	1424	0	269	—	—	3.21 [0.57, 18.05]
fostamatinib vs Aadamumab	1	1	159	0	54	—	—	1.03 [0.04, 25.70]
Malignant neoplasms by system								
Bone and articular cartilage								
fostamatinib vs PBO	2	1	765	1	354	68	0.08	0.36 [0.02, 7.67]
ill-defined, secondary and unspecified sites								
fostamatinib vs PBO	3	2	1222	1	759	74	0.05	1.58 [0.16, 15.90]
fostamatinib 100 mg bid vs. 150 mg qd	4	6	2719	0	1111	0	0.99	4.64 [0.80, 26.88]
Digestive organs								
fostamatinib 100 mg bid vs. 150 mg qd	2	1	1957	3	661	0	0.92	**0.06 [0.01, 0.59]**
urinary tract								
fostamatinib vs PBO	4	4	1273	0	613	0	1	4.33 [0.52, 36.07]
fostamatinib 100 mg bid vs. 150 mg qd	5	2	2263	2	968	50	0.11	0.60 [0.08, 4.81]
Benign neoplasms								
Fostamatinib vs PBO	6	4	2038	1	967	0	0.74	1.71 [0.26, 11.36]
12 weeks	1	0	142	0	47	—	—	—
24 weeks	2	1	372	0	161	—	—	3.77 [0.04, 356.08]
26 weeks	1	1	304	0	153	—	—	4.50 [0.07, 286.14]
52 weeks	2	2	1220	1	606	0	0.39	0.99 [0.09, 11.00]
Fostamatinib 100 mg bid vs. 150 mg qd	7	6	2954	5	1339	0	0.53	0.41 [0.10, 1.57]
12 weeks	1	0	49	0	47	—	—	—
24 weeks	2	0	186	1	181	—	—	0.12 [0.00, 6.15]
26 weeks	1	0	152	1	152	—	—	0.14 [0.00, 6.82]
52 weeks	2	1	1224	1	602	55	0.13	0.46 [0.02, 8.82]
109 weeks	1	5	1343	2	357	—	—	0.63 [0.10, 3.91]
fostamatinib 100 mg bid vs. 100 mg qd	2	5	1424	0	269	—	—	3.19 [0.25, 41.20]

Bold values indicate statistical significance.

For fostamatinib dosages of 100 mg bid vs. 150 mg qd, seven trials reported 25 neoplasms in 2,954 RA patients treated with 100 mg of fostamatinib twice daily; 100 mg fostamatinib twice daily did not increase the neoplasm risk compared with 150 mg fostamatinib use once daily for 12 neoplasms in 1,339 RA patients (Peto OR = 0.81, 95%CI 0.39–1.69, and I^2^ = 35). Similarly, there was no difference in the neoplasm risk between fostamatinib dosages of 100 mg twice daily and 150 mg once daily, with longer durations of fostamatinib use (Table 2; Supplementary Figure S1).

For fostamatinib dosages of 100 mg bid vs. 100 mg qd, two studies were concerned with the neoplasm risk in RA patients treated with these different usage times of fostamatinib. A dosage timing of twice daily did not have a higher neoplasm risk compared with 100 mg of fostamatinib once daily (Peto OR = 3.22, 95%CI 0.77–13.52, 16/1424 vs. 0/269) (Table 2; Supplementary Figure S2).

#### 3.5.2 Malignant Neoplasms

When comparing fostamatinib and placebo, six trials reported 12 malignant neoplasms in 2038 RA patients treated with fostamatinib, which did not increase the neoplasm risk compared with the placebo in one malignant neoplasm in 967 RA patients (Peto OR = 3.08, 95%CI 0.96–9.91, and I^2^ = 6) (Table 2; Supplementary Figure S3). Nevertheless, the longer use of fostamatinib had a higher malignant neoplasm risk than the placebo (Peto OR = 4.49, 95%CI 1.03–19.60, 8/1220 vs. 0/606 for 52 weeks, I^2^ = 0) (Table 2, Supplementary Figure S3).

When comparing different doses of fostamatinib, compared with 150 mg of fostamatinib once daily, 100 mg of fostamatinib twice daily did not have a higher malignant neoplasm risk (Peto OR = 1.09, 95%CI 0.45–2.60, I^2^ = 39, 19/2954 vs. 7/1339, *n* = 7) (Table 2, Supplementary Figure S4). In comparison with 100 mg of fostamatinib once daily, 100 mg of fostamatinib twice daily did not increase the malignant neoplasm risk (Peto OR = 3.21, 95%CI 0.57–18.05, and 11/1424 vs. 0/269, and *n* = 2) (Table 2, Supplementary Figure S5).

Regarding fostamatinib vs. Adamumab, one trial examined the differences in the malignant neoplasm risk, and the use of fostamatinib did not have a higher malignant neoplasm risk compared to adamumab (Peto OR = 1.03, 95%CI 0.04–25.70, and 1/159 vs. 0/54) (Table 2, Supplementary Figure S6).

#### 3.5.3 Malignant Neoplasms by System

For the bone and articular cartilage system, one malignant neoplasm was reported in 765 RA patients treated with fostamatinib, which was comparable to that in RA patients treated with placebo (Peto OR = 0.36, 95%CI 0.02–7.67, 1/765 vs. 1/354, I^2^ = 68, and *n* = 2) (Table 2; Supplementary Figure S7).

For ill-defined, secondary, and unspecified sites, four studies reported malignant neoplasms. Compared to placebo, fostamatinib did not have a higher malignant neoplasm risk than placebo (Peto OR = 1.58, 95%CI 0.16–15.90, 2/1222 vs. 1/759, I^2^ = 74, and n = 3) (Table 2; Supplementary Figure S8). A dosage of 100 mg of fostamatinib twice daily did not increase the risk of malignant neoplasms compared to 150 mg of fostamatinib once daily (Peto OR = 4.64, 95%CI 0.80–26.88, 6/2719 vs. 0/1111, I^2^ = 0, and *n* = 4) (Table 2; Supplementary Figure S9).

For the digestive organs, two trials reported malignant neoplasms. RA patients who used fostamatinib 100 mg twice daily had a lower risk of malignant neoplasms than those who used 150 mg of fostamatinib once daily (Peto OR = 0.06, 95%CI 0.01–0.59, 1/1957 vs. 3/661, I^2^ = 0) (Table 2; Supplementary Figure S10).

For the urinary tract, there were five trials focused on malignant neoplasms. The use of fostamatinib did not have a higher risk of malignant neoplasms than the placebo (Peto OR = 4.33, 95%CI 0.52–36.07, 4/1273 vs. 0/613, I^2^ = 0, and *n* = 4) (Table 2; Supplementary Figure S11). RA patients who used fostamatinib 100 mg twice daily did not have an increased risk of malignant neoplasms compared to those who used 150 mg of fostamatinib once daily (Peto OR = 0.60, 95%CI 0.08–4.81, 2/2263 vs. 2/968, I^2^ = 50, and *n* = 5) (Table 2; Supplementary Figure S12).

#### 3.5.4 Benign Neoplasms

Compared to placebo, the use of fostamatinib did not have a higher benign neoplasm risk (Peto OR = 1.71, 95%CI 0.26–11.36, I^2^ = 0, 4/2038 vs. 1/967, and *n* = 6) (Table 2, Supplementary Figure S13). A longer duration of fostamatinib use did not increase the risk of benign neoplasms (Table 2; Supplementary Figure S13). Compared to 150 mg of fostamatinib once daily, 100 mg of fostamatinib twice daily did not elevate the risk of benign neoplasms (Peto OR = 0.41, 95%CI 0.10–1.57, I^2^ = 0, 6/2954 vs. 5/1339, and *n* = 7) (Table 2; Supplementary Figure S14). Similarly, long-term use of 100 mg of fostamatinib twice daily did not increase the risk of benign neoplasms (Table 2; Supplementary Figure S14).

In total, two studies reported a benign neoplasm risk in RA patients treated with different dosage times of fostamatinib. For 100 mg of fostamatinib, a dosage time of twice daily did not increase benign neoplasm risk compared to once daily (Peto OR = 3.19, 95%CI 0.25–41.20, 5/1424 vs. 0/269) (Table 2; Supplementary Figure S15).

## 4 Discussion

### 4.1 Main Findings

Our results indicate that fostamatinib was not associated with the risks of overall neoplasms as compared to placebo (16 cases in 2038 participants vs 2 cases in 967 participants), whereas, use of fostamatinib might be related to increased malignant neoplasm risk: longer duration of fostamatinib use might be correlated with an increased risk of malignant neoplasms, 8 cases in 1220 participants vs 0 cases in 606 participants at 52 weeks, and higher dose of fostamatinib may increase the malignant neoplasm risk in the digestive system, 3 cases in 661 participants who used 150 mg once a day vs 1 case in 1957 participants taking 100 mg twice a day.

Askling et al. reported that the highest crude incidence rates of all malignancies excluding non-melanoma skin cancer, solid malignancies, all skin cancers, and malignant lymphomas in RA patients treated with fostamatinib were 1.36, 1.47, 0.74, and 0.10 per 100 person-years, respectively (Askling et al., 2016), which were similar in RA patients with other treatments. The incidence of solid cancer in RA patients treated with tumor necrosis factor α inhibitors (TNFi) and conventional synthetic disease-modifying anti-rheumatic drugs (csDMARDs) was 0.81 and 1.17 per 100 person-years, respectively, and no difference was found between the two groups after adjusting for baseline characteristics (hazard ratio [HR] = 0.83, 95%CI 0.64–1.07) (Mercer et al., 2015).

Although the high inflammatory activity of RA has been reported to be associated with increased lymphoma (Baecklund et al., 2006), Simon et al. suggested that some types of malignant tumors are related to treatment rather than the underlying disease (Simon et al., 2015). However, compared with no treatment, treatments such as those involving TNFi (incidence rate ratios [IRR] = 1.1, 95%CI 0.8–1.6) and tocilizumab (IRR = 1.2, 95%CI 0.5–2.9) were not associated with increased melanoma risk (Mercer et al., 2017). Further, compared with csDMARD, TNFi did not increase the risk of lymphomas, solid cancers excluding non-melanoma skin cancer, non-melanoma skin cancer, and melanoma skin cancer in patients with RA (Chen et al., 2016; De Cock and Hyrich, 2018).

By targeting Syk, fostamatinib has been used to treat a wide range of diseases, such as graft-versus-host disease (Flynn et al., 2015), follicular lymphoma (Fruchon et al., 2012), chronic lymphocytic leukemia (Quiroga et al., 2009), Waldenström macroglobulinemia (Kuiatse et al., 2015), ulcerative colitis (Can et al., 2015), and idiopathic thrombocytopenic purpura (Bajpai, 2009), in addition to treating RA. The mechanisms underlying the treatment of such diseases include targeting Syk signaling in B-cells and promoting their apoptosis for graft-versus-host disease (Flynn et al., 2015); suppressing the expression of matrix metalloproteinase 9 and angiogenesis through Syk-mTOR pathway for follicular lymphoma (Fruchon et al., 2012); inhibiting the phosphorylation of B-cell downstream signaling molecules, Syk, ERK, and AKT to reduce the production of CXCL12 and CXCL13 chemokines for chronic lymphocytic leukemia (Quiroga et al., 2009); inhibiting the activation of Syk and Bruton’s tyrosine kinase and suppressing downstream signaling through MAPK kinase (MEK), p44/42 MAPK, and protein kinase B/Akt to prolong the onset of tumor growth and reduce viability of primary Waldenström macroglobulinemia cells (Kuiatse et al., 2015); inhibiting tissue myeloperoxidase activity and suppressing the molecular expressions of TNFα, CD3, Syk, and phospho-Syk in tissues (Can et al., 2015); reducing inflammation through decreased major inflammatory cytokines such as TNFα, IL-1, and IL-6 and inhibiting bone degradation for the autoimmune diseases idiopathic thrombocytopenic purpura and RA (Bajpai, 2009; Boccia et al., 2020).

Moreover, fostamatinib can reduce inflammatory cell adhesion and migration, diminish macrophage survival, and normalize upregulated monocytosis and inflammatory gene expression induced by a high-cholesterol diet (Hilgendorf et al., 2011). Thus, fostamatinib can be used to treat RA. Theoretically, fostamatinib can reduce the disease activity of RA and be used to treat lymphoma; thus, the finding of this study that longer-time use of fostamatinib could increase malignant neoplasm risk should be explained with caution. Fostamatinib is only effective in approximately 50% (680/1419) of RA patients assessed by ACR20, (Kunwar et al., 2016); therefore, uncontrolled disease activity would contribute to the risk of malignant tumors. In contrast, owing to the limited data, subgroup analyses were only performed for the nature of the neoplasm, follow-up periods, and the neoplasm-originating system, but analysis of subtypes of malignant neoplasms was not conducted. Thus, we could not focus on a specific neoplasm type, such as B-cell lymphoma, as Syk is reported to be necessary for B-cell development, proliferation, and survival.

## Limitations

Several limitations of this meta-analysis and systematic review should be considered. First, only RCTs were eligible for inclusion, whereas no cohort and case-control studies were included. Although RCTs can balance the baseline measurements, having the least potential bias and less likely to be affected by possible confounders, the sample size and follow-up duration were relatively small, and short. For the small smaple size, the minimal reported sample size in the subgroup in our review was only based on 96 participants. Especially for rare cases, such as neoplasm, a small sample size may be underpowered to detect the outcomes. Moreover, the number of included studies was small and only seven RCTs were included. When performing subgroup analysis, several outcomes were reported only by one study; thus, the confidence intervals were relatively wide, affecting the reliability of the results. Therefore, well-planned observational studies with large study populations, such as cohort studies, are needed to determine whether fostamatinib is associated with increased or decreased malignant neoplasm risk in RA patients.

## Conclusion

Our findings suggest that a longer duration of fostamatinib use in RA patients increases the risk of malignant neoplasms and a higher dose of fostamatinib may increase malignant neoplasms in the digestive system. However, owing to the small sample size and short follow-up duration, further studies such as cohort studies with large study populations and longer follow-up times are required to rule out the results.

## Data Availability

The original contributions presented in the study are included in the article/Supplementary Material, further inquiries can be directed to the corresponding authors.
