# Supplementary material for: Neoplasm Risk in Patients With Rheumatoid Arthritis Treated With Fostamatinib: A Systematic Review and Meta-analysis
Source: Front Pharmacol. 2022 Mar 2;13:768980. doi: 10.3389/fphar.2022.768980 (PMC8926144; doi:10.3389/fphar.2022.768980)
Supplement: Supplementary file 2 [file DataSheet2.docx]

**Neoplasm risk in patients with rheumatoid arthritis treated with fostamatinib: a systematic review and meta-analysis**

Yuehong Chen, Huan Liu, Yunru Tian, Zhongling Luo, Geng Yin, Qibing Xie

Department of Rheumatology and Immunology, West China Hospital, Sichuan University, Chengdu 610041, China

**Search Strategy:**

**Search date: July 3, 2020**

**Database: Ovid MEDLINE(R) <1946 to June Week 4 2020> (n=118)**

1. “rheumatoid arthritis.mp. ” OR “exp Arthritis, Rheumatoid/” OR “((rheumatoid or reumatoid or revmatoid or rheumatic or reumatic or revmatic or rheumat$ or reumat$ or revmarthrit$) adj3 (arthrit$ or artrit$ or diseas$ or condition$ or nodule$)).tw.” OR “(felty$ adj2 syndrome).tw.” OR “(caplan$ adj2 syndrome).tw.” OR “(sjogren$ adj2 syndrome).tw.” OR “(sicca adj2 syndrome).tw.” OR “still$ disease.tw.” OR “bechterew$ disease.tw.”
2. “Fostamatinib.mp.” OR “Fostamatinib Disodium.mp.” OR “Syk Kinase/” OR

“Tavalisse.mp.” OR “Masitinib.mp.” OR “AB1010.mp.” OR “ab1010.mp.” OR “AB 1010.mp.” OR “ab 1010.mp.” OR “AB-1010.mp.” OR “UNII-M59NC4E26P.mp.” OR “R-788.mp.” OR “22 r-788.mp.” OR “R 788.mp.” OR “R788.mp.” OR “tamatinib fosdium.mp.” OR “R935788.mp.” OR “R 935788.mp.” OR “R-935788.mp.” OR “R-788$400.mp.” OR “R 788$400.mp.” OR “R788$400.mp.”

1. #1 AND #2

**Database: Embase <1974 to 2020 July 02> (n=269)**

1. “rheumatoid arthritis.mp.” OR “exp rheumatoid arthritis/” OR “((rheumatoid or reumatoid or revmatoid or rheumatic or reumatic or revmatic or rheumat$ or reumat$ or revmarthrit$) adj3 (arthrit$ or artrit$ or diseas$ or condition$ or nodule$)).tw.” OR “(felty$ adj2 syndrome).tw.” OR “(caplan$ adj2 syndrome).tw.” OR “(sjogren$ adj2 syndrome).tw.” OR “(sicca adj2 syndrome).tw.” OR “still$ disease.tw.” OR “bechterew$ disease.tw.”

2. “Fostamatinib.mp.” OR “exp fostamatinib/” OR “Fostamatinib Disodium.mp.” OR “Tavalisse.mp.” OR “Masitinib.mp.” OR “AB1010.mp.” OR “ab1010.mp.” OR “AB 1010.mp.” OR “ab 1010.mp.” OR “AB-1010.mp.” OR “UNII-M59NC4E26P.mp.” OR “R-788.mp.” OR “r-788.mp.” OR “R 788.mp.” OR “R788.mp.” OR “tamatinib fosdium.mp.” OR “R935788.mp.” OR “R 935788.mp.” OR “R-935788.mp.” OR “R-788-400.mp.” OR “R-788$400.mp.” OR “R 788$400.mp.” OR “R788$400.mp.”

3. #1 AND #2

**Database: Web of science (1900-2020) (n=127)**

1. “TS=(rheumatoid arthritis)” OR“ALL=((rheumatoid or reumatoid or revmatoid or rheumatic or reumatic or revmatic or rheumat$ or reumat$ or revmarthrit$) adj3 (arthrit$ or artrit$ or diseas$ or condition$ or nodule$)).tw.)”

2. “TS=(Fostamatinib)” OR “TS=(Fostamatinib Disodium)” OR “ALL=(Fostamatinib)” OR “ALL=(Tavalisse)” OR “ALL=(Masitinib)” OR “ALL=(AB1010)” OR “ALL=(AB 1010)” OR “ALL=(AB-1010)” OR “ALL=(R-788)” OR “ALL=(R 788)” OR “ALL=(R788)” OR “ALL=(tamatinib fosdium)” OR “ALL=(Fostamatinib Disodium)” OR “ALL=(R935788)” OR “ALL=(R 935788)” OR “ALL=( R-935788)” OR “ALL=( R-788-400)” OR “ALL=(R-788$400)” OR “ALL=(R 788$400)” OR “ALL=(R788$400)”

3. #1 AND #2

**Cochrane library (n=44)**

1. “MeSH descriptor: [Arthritis, Rheumatoid] explode all trees” OR “(rheumatoid arthritis):ti,ab,kw” OR “(felty$ adj2 syndrome):ti,ab,kw” OR “(caplan$ adj2 syndrome):ti,ab,kw” OR “(sjogren$ adj2 syndrome):ti,ab,kw” OR “(sicca adj2 syndrome):ti,ab,kw” OR “still$ disease:ti,ab,kw” OR “bechterew$ disease:ti,ab,kw”

2. “(Fostamatinib):ti,ab,kw” OR “(Fostamatinib Disodium):ti,ab,kw” OR “(Tavalisse):ti,ab,kw” OR “(Masitinib):ti,ab,kw” OR “(AB1010):ti,ab,kw” OR “(ab1010):ti,ab,kw” OR “(AB 1010):ti,ab,kw” OR “(ab 1010):ti,ab,kw” OR “(AB-1010):ti,ab,kw” OR “(R-788):ti,ab,kw” OR “(r-788):ti,ab,kw” OR “(R 788):ti,ab,kw” OR “(R788):ti,ab,kw” OR “(tamatinib fosdium):ti,ab,kw” OR “(R935788):ti,ab,kw” OR “(R 935788):ti,ab,kw” OR “(R-935788):ti,ab,kw” OR “(R-788-400):ti,ab,kw” OR “(R-788$400):ti,ab,kw” OR “(R 788$400):ti,ab,kw” OR

“(R788$400):ti,ab,kw”

3.#1 NAD #2
