# Supplementary material for: Neoplasm Risk in Patients With Rheumatoid Arthritis Treated With Fostamatinib: A Systematic Review and Meta-analysis
Source: Front Pharmacol. 2022 Mar 2;13:768980. doi: 10.3389/fphar.2022.768980 (PMC8926144; doi:10.3389/fphar.2022.768980)
Supplement: Supplementary file 3 [file DataSheet1.docx]

**Neoplasm risk in patients with rheumatoid arthritis treated with fostamatinib: a systematic review and meta-analysis**

Yuehong Chen, Huan Liu, Yunru Tian, Zhongling Luo, Geng Yin, Qibing Xie

Department of Rheumatology and Immunology, West China Hospital, Sichuan University, Chengdu 610041, China

**Supplemental figures**

sFig.1-sFig.15 page 2-page 8


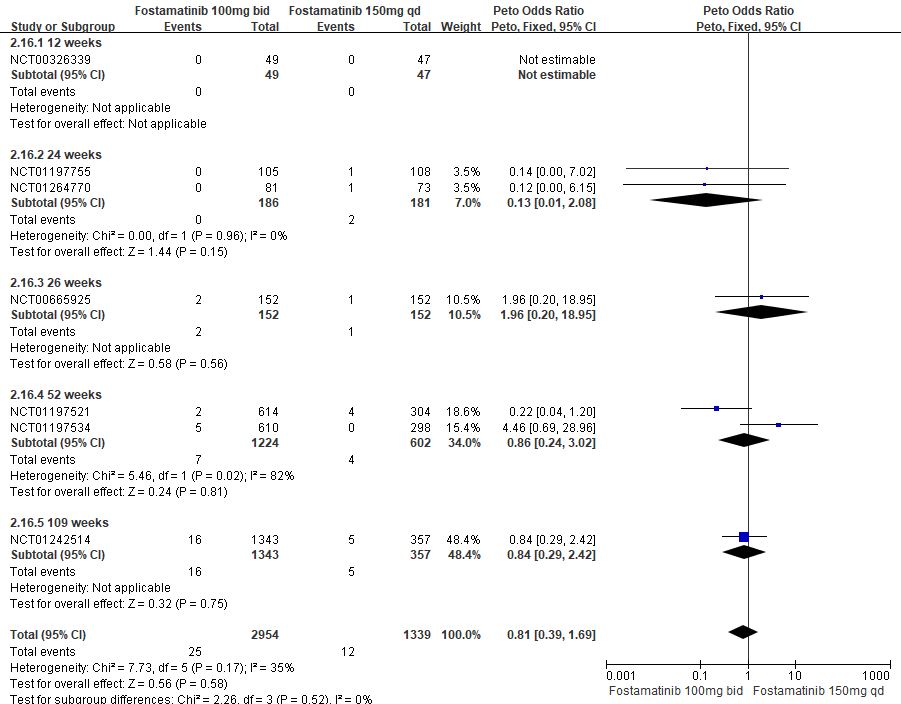


sFig.1 Forest plot of overall neoplasms based on fostamatinib 100mg bid versus 150mg qd.


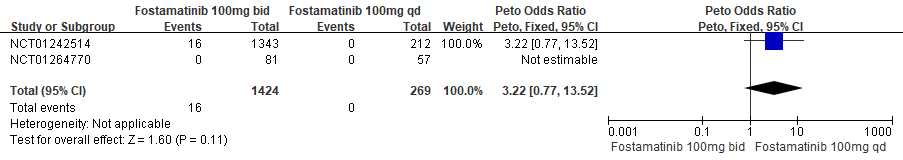


sFig.2 Forest plot of overall neoplasms based on fostamatinib 100mg bid versus 100mg qd.


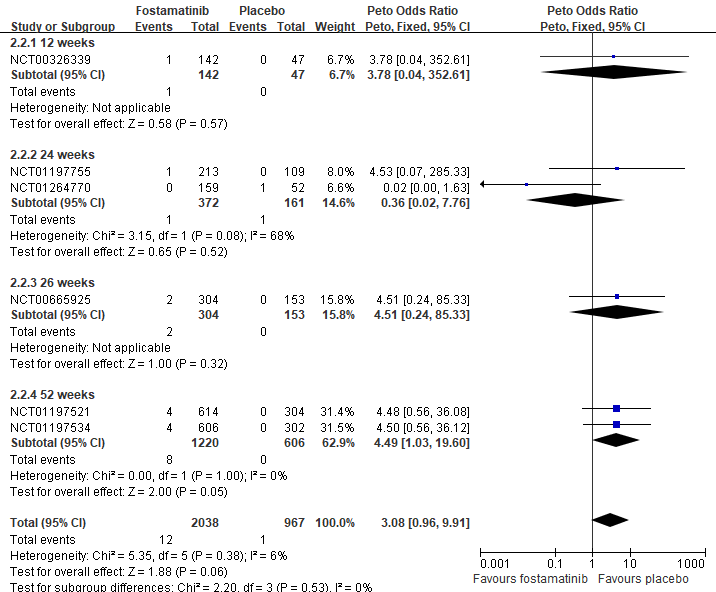


sFig.3 Forest plot of malignant neoplasms based on fostamatinib versus placebo.


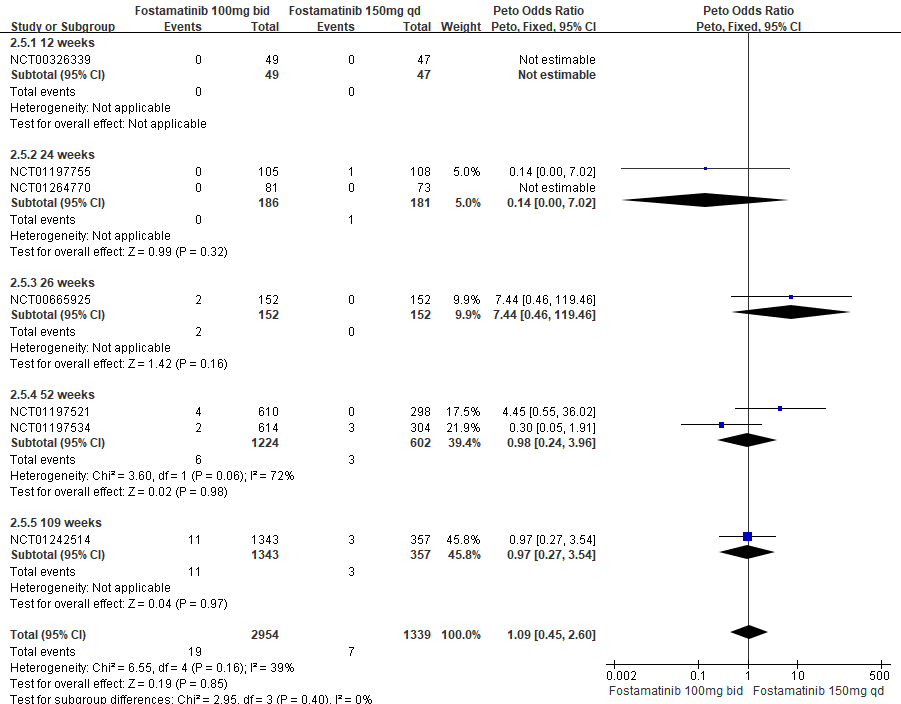


sFig.4 Forest plot of malignant neoplasms based on fostamatinib 100mg bid versus 150mg qd.


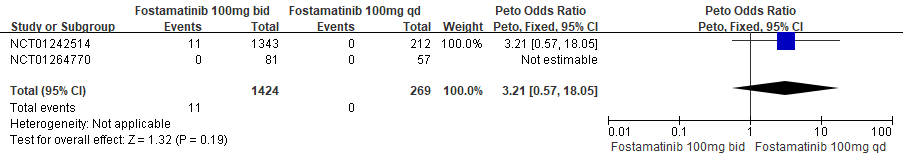


sFig.5 Forest plot of malignant neoplasms based on fostamatinib 100mg bid versus 100mg qd.


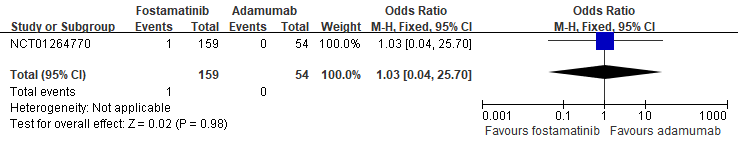


sFig.6 Forest plot of malignant neoplasms based on fostamatinib versus adamumab.


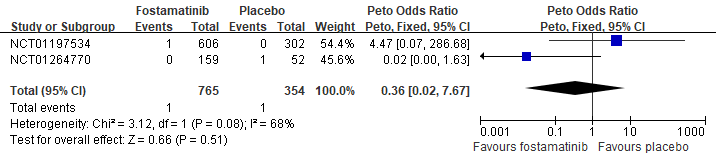
sFig.7 Forest plot of malignant neoplasms at bone and articular cartilage (C40-C41) based on fostamatinib versus placebo


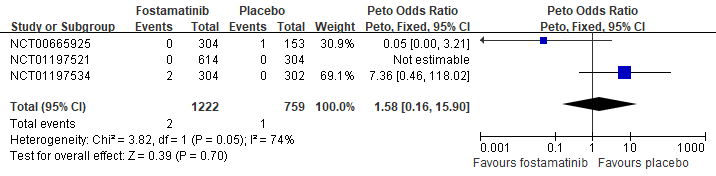


sFig.8 Forest plot of malignant neoplasms at ill-defined, secondary and unspecified sites (C76-C80) based on fostamatinib versus placebo.


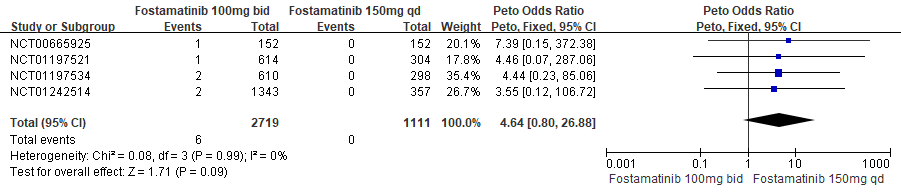


sFig.9 Forest plot of malignant neoplasms at ill-defined, secondary and unspecified sites (C76-C80) based on fostamatinib 100mg bid versus 150mg qd.


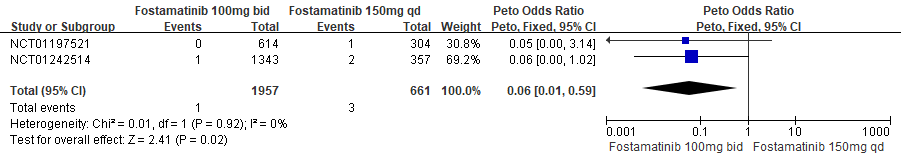


sFig.10 Forest plot of malignant neoplasms of digestive organs (C15-C26) based on fostamatinib 100mg bid versus 150mg qd.


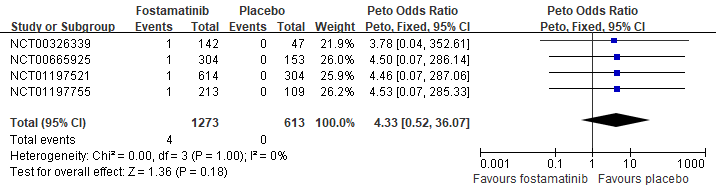


sFig.11 Forest plot of malignant neoplasms of urinary tract (C64-C68) based on fostamatinib versus placebo.


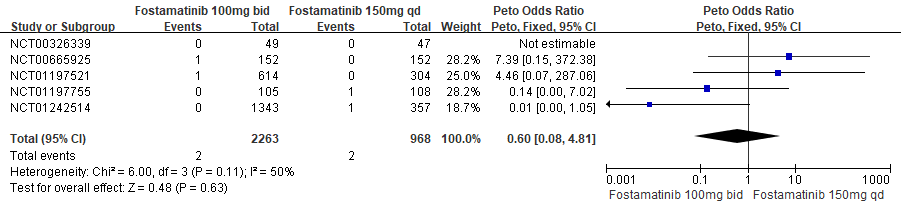


sFig.12 Forest plot of malignant neoplasms of urinary tract (C64-C68) based on fostamatinib 100mg bid versus 150mg qd.


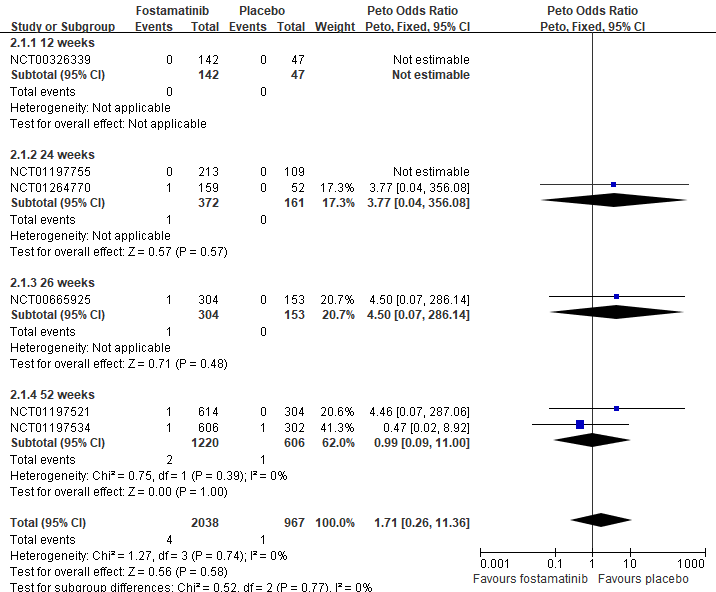


sFig.13 Forest plot of benign neoplasms based on fostamatinib versus placebo.


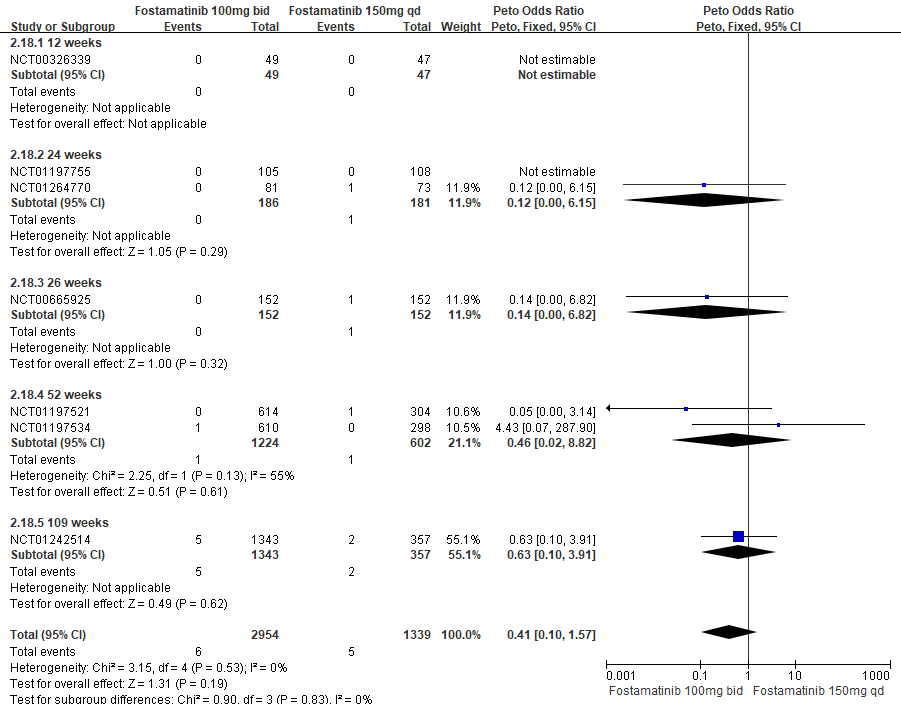


sFig.14 Forest plot of benign neoplasms based on fostamatinib 100mg bid versus fostamatinib 150mg qd.


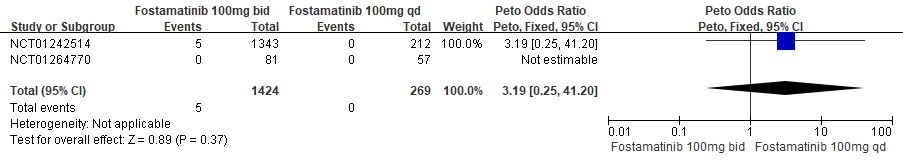


sFig.15 Forest plot of benign neoplasms based on fostamatinib 100mg bid versus fostamatinib 100mg qd.
